# Neuregulin-1-mediated ErbB2–ErbB3 signalling protects human trophoblasts against apoptosis to preserve differentiation

**DOI:** 10.1242/jcs.176933

**Published:** 2015-12-01

**Authors:** Valerie Fock, Kerstin Plessl, Peter Draxler, Gerlinde Regina Otti, Christian Fiala, Martin Knöfler, Jürgen Pollheimer

**Affiliations:** 1 Department of Obstetrics and Fetal-Maternal Medicine, Reproductive Biology Unit, Medical University of Vienna, Vienna 1090, Austria; 2 Department of Neurophysiology, Center for Brain Research, Medical University of Vienna, Vienna 1090, Austria; 3 Gynmed Clinic, 1150 Vienna, Austria

**Keywords:** Trophoblast, Neuregulin 1, ErbB2, ErbB3, Apoptosis, Differentiation

## Abstract

During placentation, foetal trophoblasts invade deeply into maternal tissue to establish a foeto–maternal circulation. We have previously shown that extravillous trophoblast (EVT) lineage cells express ErbB2 and ErbB3, of which the potential as an oncogenic unit is well established. However, a physiological function of this receptor combination in humans remains a puzzling question. Here, we demonstrate neuregulin 1 (NRG1) expression and secretion by human decidual stromal cells. Stimulation of human primary trophoblasts with exogenous NRG1 induced phosphorylation of ErbB2, ErbB3 and related downstream effectors. Co-immunoprecipitation experiments confirmed the formation of ErbB2–ErbB3 dimers upon ligand engagement. Along this line, receptor knockdown and ErbB3 neutralization strongly diminished NRG1-dependent activation of the signalling complex. Functional studies revealed that NRG1 promotes EVT formation in placental explant cultures. Although, in the presence of NRG1, basal and camptothecin-induced trophoblast apoptosis was significantly repressed, this effect was abolished upon ErbB3 inhibition. Notably, camptothecin provoked a strong reduction of trophoblast cell column size, whereas NRG1-treated explants were refractory to the compound. Taken together, our findings newly identify a physiological function of the NRG1–ErbB2–ErbB3 axis in trophoblast survival during human placental development.

## Abstract (precis)

**Highlighted Article:** Decidual-stromal-cell-derived neuregulin-1 induces ErbB2–ErbB3 heterodimerization in extravillous trophoblasts and stabilizes trophoblast differentiation through suppression of apoptosis.

## INTRODUCTION

Neuregulin 1 (NRG1) is widely known to play an essential role in the heart and nervous systems, where it orchestrates vital cell functions such as mitogenesis and differentiation (Meyer and Birchmeier, 1995). However, emerging evidence suggests the involvement of NRG1 signalling in the development and function of other organ systems as well as in the pathogenesis of breast cancer and schizophrenia (Falls, 2003).

NRG1 mediates its effects by binding to members of the erythroblastoma (ErbB) family, consisting of epidermal growth factor receptor (EGFR)/ErbB1, ErbB2, ErbB3 and ErbB4. The latter two represent the bona fide receptors for NRG1 (Mei and Xiong, 2008). Upon ligand engagement, ErbB receptors undergo homo- or heterodimerization, which is followed by auto- and trans-phosphorylation of intracellular tyrosine residues and the recruitment of adaptor proteins. The phosphatidylinositide 3-kinase (PI3K)–protein-kinase-B (Akt) pathway as well as the mitogen-activated protein kinase (MAPK) cascade frequently participate in ErbB signalling to govern distinct cell fate decisions (Citri and Yarden, 2006). ErbB3 is a non-autonomous receptor with an impaired tyrosine kinase domain, and thus homodimers are catalytically inactive (Guy et al., 1994). The ligand-less ErbB2 receptor always exists in an untethered conformation, poised to dimerize with another receptor and therefore acts as the preferred heterodimerization partner of all other family members (Garrett et al., 2003).

Remarkably, ErbB2–ErbB3 heterodimers represent the most potent signalling complexes and function as an oncogenic unit that has been implicated in cell cycle progression, survival, angiogenesis and metastasis (Ursini-Siegel et al., 2007). To date, a physiological role of ErbB2–ErbB3 signalling has only been described in murine neurons (Britsch et al., 1998). A recent publication by our laboratory identifies human trophoblast subtypes that specifically express ErbB2 and ErbB3, while being devoid of EGFR and ErbB4 (Fock et al., 2015). These data point to a physiological function of the NRG1–ErbB2–ErbB3 axis during placental development in humans.

Trophoblasts are specialized cells of the placenta and play a crucial role in implantation, in the supply of nutrients to the foetus and in the immune tolerance of the foetus in an allogeneic environment (Moffett and Loke, 2006). Placental villi, the basic structural units of the human placenta, comprise an outer syncytiotrophoblast and an inner villous cytotrophoblast (CTB) layer surrounding the stromal core. In the course of placentation, villous CTBs give rise to cells of the extravillous trophoblast (EVT) lineage, which infiltrate the decidualized endometrium and remodel local arteries to establish a foeto–maternal circulation (Pijnenborg et al., 1980). EVT formation is initiated by increased villous CTB proliferation, leading to the emergence of multilayered cell columns at the tips of anchoring villi contacting the decidua. At their very proximal end, cell columns comprise proliferative EGFR^+^ cell column trophoblasts (CCTs), which continuously differentiate into non-dividing trophoblasts, forming the distal part of nascent columns. The latter acquire an invasive phenotype, characterized by the upregulation of specific marker genes such as human leukocyte antigen (HLA)-G (Ellis et al., 1990), T-cell factor (TCF)4 (Pollheimer et al., 2006), integrin α (ITGA)1 and ITGA5 (Damsky et al., 1992), as well as various matrix metalloproteinases (MMPs) (Fisher et al., 1989).

The development of the EVT lineage is tightly controlled by a myriad of factors, and failure of this process is associated with severe pregnancy disorders, including pre-eclampsia and foetal growth restriction (Brosens et al., 1977, 1970). In light of the manifold cellular functions that are modulated by the ErbB network, we hypothesized that ErbB2 and ErbB3 might represent important regulators of EVT formation. Hence, it was the scope of the present study to unravel functional aspects of this oncogenic receptor combination during human placental development.

## RESULTS

### Decidua-derived signals activate ErbB3 and related downstream kinases in primary trophoblasts

**Fig. 1.**
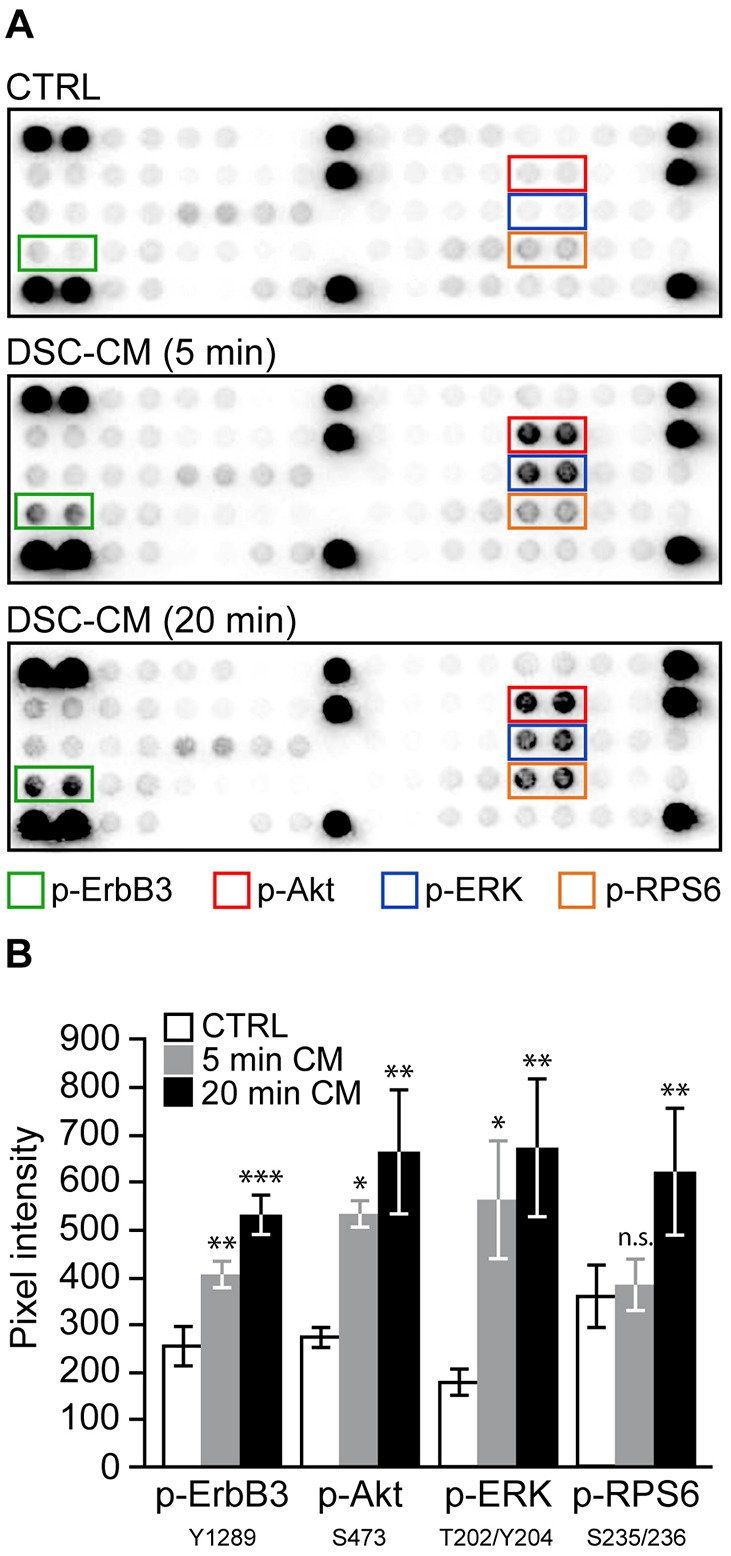
**Treatment of primary trophoblasts with DSC-conditioned medium triggers phosphorylation of ErbB3 and downstream signalling mediators.** (A) Serum-free conditioned medium (CM) was harvested from overnight-cultivated decidual stromal cells (DSCs). Primary trophoblasts were starved for 4 h and stimulated with DSC-CM for 5 and 20 min. Control (CTRL) treatment: serum-free culture medium. Screening of phosphorylated proteins was performed using the PathScan^®^ RTK Signaling Antibody Array Kit. Representative examples of each condition are shown. p-, phosphorylated protein. (B) Pixel intensities were quantified by using densitometry analysis using Alpha View software. Data are presented as mean±s.d. of three independent experiments. Statistical significance was determined by a one-way ANOVA followed by Bonferroni's post-hoc correction. *P<0.05, **P<0.01, ***P<0.001; n.s., not significant.

We have recently shown that EVT formation is accompanied by the acquisition of a specific receptor tyrosine kinase (RTK) signature, including fibroblast growth factor receptors FGFR1 and FGFR3, Fms-related tyrosine kinases FLT1 and FLT4, as well as ErbB2 and ErbB3 (Fock et al., 2015). Given the close spatial relationship between the EVT lineage and cells within the decidua, we reasoned that signalling molecules derived from maternal tissue influences the activation status of these receptors in a paracrine manner. To this end, we stimulated primary trophoblasts with conditioned medium harvested from overnight-cultivated decidual stromal cells (DSCs) and performed a phospho-RTK array. Indeed, ErbB3 was strongly phosphorylated at tyrosine residues in the presence of DSC-conditioned medium, whereas the activation status of other receptors remained unaffected. Furthermore, pronounced phosphorylation of the downstream effectors Akt1 at residue S473, extracellular signal-regulated kinases 1 and 2 (also known as MAPK3 and MAPK1, respectively; ERK1/2) at residues T202 and Y204, and ribosomal protein S6 (RPS6) at residues S235 and S236 could be observed (Fig. 1A). Measurement of pixel intensities revealed significantly increased levels of phosphorylated ErbB3, Akt1, ERK1/2 and RPS6 after 5 and/or 20 min of stimulation (Fig. 1B).

### NRG1 is released by DSCs, and triggers phosphorylation of ErbB2 and ErbB3 in primary trophoblasts

**Fig. 2.**
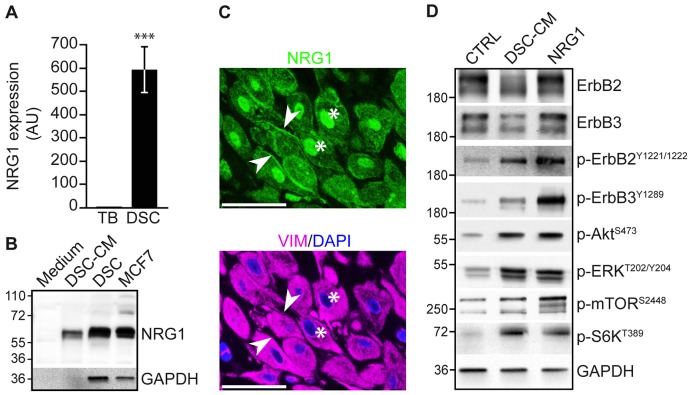
**NRG1 is produced by the decidua and induces a phosphorylation pattern in trophoblasts akin to that with DSC-conditioned medium.** (A) *NRG1* mRNA expression of primary trophoblasts (TBs) and decidual stromal cells (DSCs) was assessed by using RT-PCR analysis. Data are presented as mean±s.d. relative to TB (arbitrarily set to 1) of three independent experiments. Statistical significance was determined by using an unpaired two-tailed Student's *t*-test. ***P<0.001. (B) Lysates of overnight-cultivated DSCs and DSC-conditioned medium (CM) were subjected to western blot analysis to determine NRG1 protein levels. The human breast adenocarcinoma cell line MCF7 and serum-free medium were used as positive and negative controls, respectively. GAPDH served as loading control. (C) Immunofluorescent co-staining for NRG1 and vimentin (VIM) were performed on first trimester decidual tissue sections (11th week). DAPI was used to visualize nuclei. Arrowheads and asterisks depict membranous and nuclear NRG1 in DSCs, respectively. Scale bars: 50 µm. (D) Primary trophoblasts were starved for 4 h and stimulated with DSC-CM or recombinant human NRG1 (20 ng/ml) for 5 min. Control (CTRL) treatment: 4 µM citrate solution. Phosphorylation of ErbB2, ErbB3 and downstream effectors was determined by western blotting. GAPDH served as loading control. Representative examples of at least three independently performed experiments are shown. p-, phosphorylated protein at the indicated residues.

In an attempt to explain the observed effects on the phosphorylation status of ErbB3, we analyzed the expression profile of the well-characterized ErbB3 ligand NRG1 in DSCs. Real-time PCR analysis revealed significantly elevated NRG1 mRNA levels in primary DSCs when compared to those of overnight-cultivated trophoblasts (Fig. 2A). Furthermore, an NRG1-specific immunoreactive band could be detected in lysates of cultivated DSCs and DSC-conditioned medium by western blotting (Fig. 2B). Immunofluorescent staining of first trimester decidual tissue confirmed NRG1 expression by vimentin^+^ DSCs. Interestingly, NRG1 localized both to the nucleus and the cell membrane (Fig. 2C). We then asked whether treatment of primary trophoblasts with recombinant human NRG1 mirrors the phosphorylation pattern observed with DSC-conditioned medium. Strikingly, we detected analogous effects of recombinant human NRG1 and DSC-derived medium on the activation status of ErbB3, Akt1, ERK1/2, mammalian target of rapamycin (mTOR) and S6 kinase (S6K) – the upstream activator of RPS6. Moreover, we noticed a marked phosphorylation of ErbB2 at residues Y1221 and Y1222 upon stimulation with recombinant human NRG1 and DSC-conditioned medium (Fig. 2D). Further analyses with antibodies recognizing two additional phosphorylation sites (Y788, Y1248) confirmed NRG1-driven activation of ErbB2 (Fig. S1A). These results indicate that decidua-derived NRG1 acts on trophoblasts in a paracrine manner to activate ErbB2, ErbB3 and downstream signal transduction pathways.

### ErbB2 and ErbB3 form a functional unit in HLA-G^+^ trophoblasts upon stimulation with NRG1

**Fig. 3.**
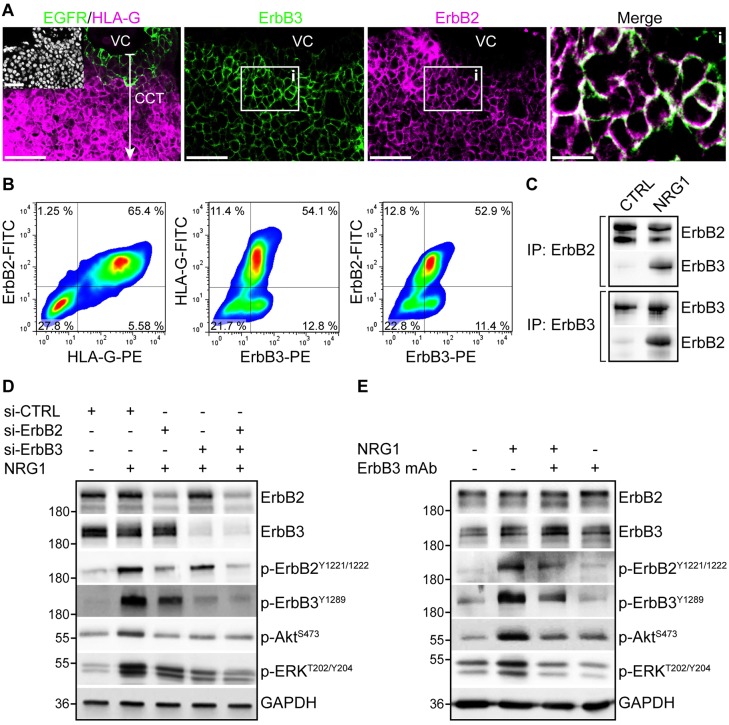
**ErbB2 and ErbB3 are co-expressed by HLA-G^+^ trophoblasts and heterodimerize upon stimulation with NRG1.** (A) Immunofluorescent co-staining for EGFR and HLA-G, or ErbB2 and ErbB3 were performed on serial sections of first trimester placental tissue (12th week). DAPI nuclear staining is depicted in the upper left corner. The merged image (i, digitally zoomed) demonstrates a significant colocalization of ErbB2 and ErbB3 (white) in HLA-G^+^ cell column trophoblasts (CCTs). VC, villous core. Scale bars: 50 µm. (B) Primary trophoblasts were co-labelled with FITC- and PE-conjugated antibodies against HLA-G, ErbB2 or ErbB3 and subjected to flow cytometry analysis. Data were analyzed using FlowJo software. The percentage of double-positive cells is displayed in the upper right quadrant. (C) Primary trophoblasts were starved for 4 h and stimulated with recombinant human NRG1 (20 ng/ml) for 5 min. Control (CTRL) treatment: 4 µM citrate solution. ErbB2 or ErbB3 was immunoprecipitated (IP) from cell lysates followed by western blotting for ErbB2 and ErbB3. (D,E) ErbB2 and ErbB3 single- and double-knockdowns (si-) were performed in primary trophoblasts (D), or cells were pre-treated with a monoclonal ErbB3-blocking antibody (ErbB3 mAb, 10 µg/ml) for 30 min (E), before being starved for 4 h and stimulated with recombinant human NRG1 (20 ng/ml) for 5 min. Control treatment: 4 µM citrate solution and 10 µg/ml IgG mAb. Phosphorylation (p-) of ErbB2, ErbB3 and downstream effectors at the indicated residues was determined by western blotting. GAPDH served as loading control. Representative examples of at least three independently performed experiments are shown.

Based on our findings, we hypothesized that ErbB2 and ErbB3 are co-expressed and able to form functional heterodimers in human trophoblasts. To test this, we performed double immunofluorescent staining on serial sections of first trimester placental tissue. Both receptors were strongly expressed by EGFR^−^/HLA-G^+^ CCTs and, indeed, showed significant colocalization in the respective trophoblast populations (Fig. 3A). In concert with our immunofluorescence data, flow cytometry analysis of primary trophoblasts confirmed that HLA-G expression precludes EGFR and ErbB4 positivity, and cells also stained double-positive for HLA-G and ErbB2 (65.4%), or HLA-G and ErbB3 (54.1%). Importantly, 52.9% of cells were double-positive for both receptors (Fig. 3B; data not shown). Furthermore, co-immunoprecipitation analyses revealed ErbB2–ErbB3 interactions in the presence of recombinant human NRG1, indicating heterodimerization of the two receptors upon ligand binding (Fig. 3C). To interfere with NRG1-mediated receptor dimerization, we transfected primary trophoblasts with siRNAs targeting ErbB2 or ErbB3 and determined their activation status. Phosphorylation of the respective receptors by recombinant human NRG1 was effectively diminished in cells that had been subjected to knockdown of one of the receptors. Accordingly, activation of the downstream effectors Akt1 and ERK1/2 was remarkably decreased (Fig. 3D). In agreement with these results, blockade of ErbB3 with a monoclonal antibody (ErbB3 mAb) led to a sustained decrease in NRG1-mediated phosphorylation of ErbB2 and ErbB3, as well as of Akt1 and ERK1/2 (Fig. 3E; Fig. S1A). Of note, the optimal concentration of the ErbB3-blocking antibody was determined by measuring dose- and time-dependent phosphorylation levels of ErbB3 and Akt1 in primary trophoblasts (Fig. S1B). Altogether, these findings strongly support the formation of signalling-competent ErbB2–ErbB3 heterodimers in differentiated HLA-G^+^ trophoblasts.

### NRG1 promotes EVT formation in placental explant cultures

**Fig. 4.**
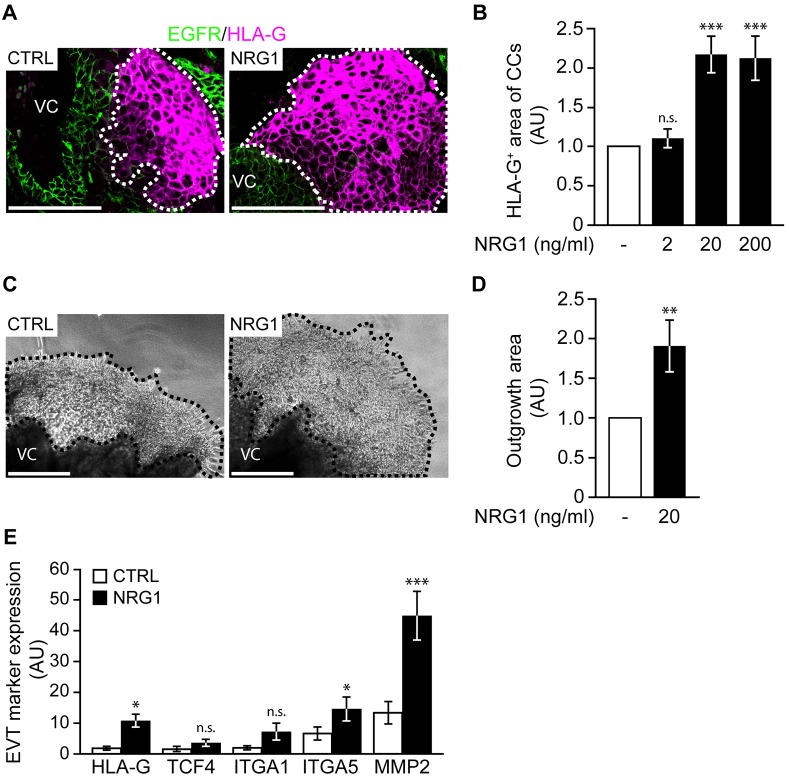
**NRG1 promotes trophoblast outgrowth and differentiation.** (A) First trimester placental floating explants were cultivated in the presence or absence of recombinant human NRG1 (20 ng/ml) for 24 h. Control (CTRL) treatment: 4 µM citrate solution. Representative immunofluorescent co-staining for EGFR and HLA-G is shown. VC, villous core. HLA-G^+^ areas of cell columns (CCs) are outlined in white. Scale bars: 50 µm. (B) The HLA-G^+^ area of cell columns was determined in the presence (black bars) or absence (white bars) of recombinant human NRG1 at the indicated concentrations using Cell^P software. (C) First trimester placental explants were cultivated on collagen-I in the presence (black bars) or absence (white bars) of recombinant human NRG1 (20 ng/ml) for 24 h. Control treatment: 4 µM citrate solution. Representative pictures of each condition are shown. VC, villous core. Scale bars: 400 µm. (D) The area of trophoblast outgrowth was determined using Cell^P software. (E) mRNA expression levels of the trophoblast differentiation markers human leukocyte antigen-G (HLA-G), T-cell factor 4 (TCF4), integrin α1 (ITGA1), ITGA5 and matrix metalloproteinase (MMP)2 in control explants and explants that had been treated with recombinant human NRG1 (20 ng/ml) were assessed by using RT-PCR. Data are expressed as mean±s.d. relative to vehicle control (B,D) or EGFR values (E) of three independent experiments. Statistical significance was determined with a one-way ANOVA test followed by Bonferroni's post-hoc correction (B), an unpaired two-tailed Student's *t*-test (D) or a two-way ANOVA followed by Šidák's post-hoc correction (E). *P<0.05, **P<0.01, ***P<0.001; n.s., not significant.

Development of the EVT lineage involves a sequential series of steps that includes cell proliferation, differentiation and invasion. These processes are mimicked by placental tissue explants forming proliferative cell columns that differentiate into invasive EVTs (Genbacev et al., 1992). To determine the biological function of NRG1-induced ErbB2–ErbB3 signalling, we first assessed the effect of NRG1 on placental floating explants that had been cultivated in serum-free medium. Interestingly, treatment with recombinant human NRG1 caused a dose-dependent increase in the HLA-G^+^ area of cell columns when compared to vehicle-treated controls (Fig. 4A,B). Additionally, we monitored the influence of recombinant human NRG1 on placental explants that had been grown on collagen-I and found that trophoblast outgrowth was significantly increased in the presence of the ligand (Fig. 4C,D). Of note, neither trophoblast cell proliferation nor invasion was affected by recombinant human NRG1 in any of the model systems used (Fig. S2). In a next step, we purified RNA from both control explants and explants that had been treated with recombinant human NRG1, and analyzed the expression levels of EVT differentiation markers by using RT-PCR analysis. A two-way ANOVA statistical test revealed a significant general effect (*P*<0.001) of recombinant human NRG1 on EVT marker expression (Fig. 4E). Further analyses using short-term recombinant human NRG1-stimulated primary trophoblasts indicated that none of these genes are direct transcriptional targets of NRG1 signalling (Fig. S3).

### NRG1 protects differentiated trophoblasts from camptothecin-induced apoptosis

**Fig. 5.**
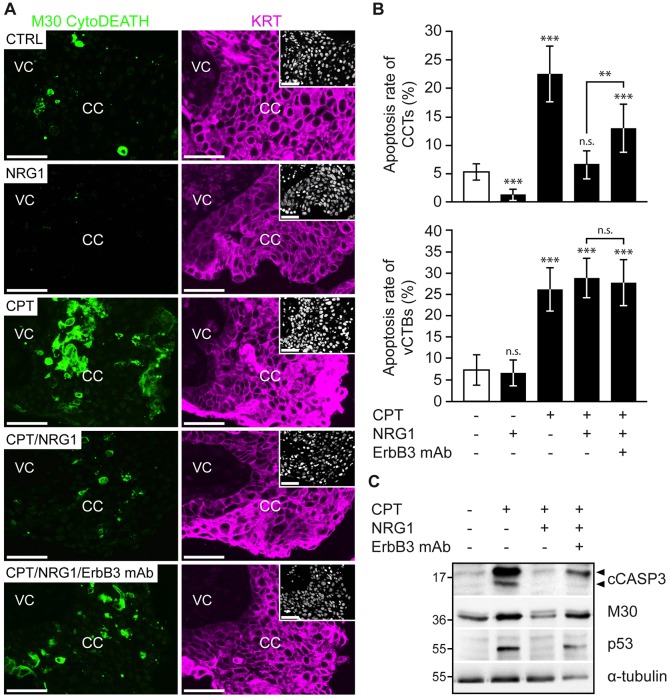
**CPT-induced trophoblast cell apoptosis is inhibited by NRG1.** (A) First trimester placental floating explants were cultivated in the presence or absence of recombinant human NRG1 (20 ng/ml) and/or camptothecin (CPT, 1 µM) for 5 h. Where indicated, explants were pre-treated with a monoclonal ErbB3-blocking antibody (ErbB3 mAb, 10 µg/ml) for 30 min. Control (CTRL) treatment: 4 µM citrate solution, 10 µM DMSO and 10 µg/ml IgG mAb. Representative immunofluorescent co-staining for M30 CytoDEATH and keratin (KRT) under each condition are shown. DAPI nuclear staining is depicted in the upper right corners. CC, cell columns; VC, villous core. Scale bars: 50 µm. (B) The rate of apoptosis was quantified as the percentage of M30^+^ nuclei relative to the number of DAPI^+^ nuclei of cell column trophoblasts (CCTs) or ErbB2^−^/ErbB3^−^ villous cytotrophoblasts (vCTBs). White bars represent untreated controls, and black bars represent NRG1-, CPT- and/or ErbB3-mAb-treated placental explants. Data are expressed as mean±s.d. of four independent experiments. Statistical significance was determined with a one-way ANOVA test followed by Bonferroni's post-hoc correction. **P<0.01, ***P<0.001; n.s., not significant. (C) Primary trophoblasts were incubated in the presence or absence of recombinant human NRG1 (20 ng/ml) and/or CPT (1 µM) for 5 h. When indicated, cells were pre-treated with an ErbB3 mAb (10 µg/ml) for 30 min. Control treatment: 4 µM citrate solution, 10 µM DMSO and 10 µg/ml IgG mAb. Activation of cleaved caspase 3 (cCASP3, arrowheads depict 17- and 19-kDa fragments), caspase-cleaved cytokeratin 18 (M30) and p53 was determined by western blotting. α-tubulin served as loading control. A representative example of three independently performed experiments is shown.

Programmed cell death has been shown to play a crucial role in placental morphogenesis and normal trophoblast function (Huppertz et al., 2006). This is also reflected in deregulated apoptosis rates in EVTs in the context of pre-eclampsia and foetal growth restriction (DiFederico et al., 1999; Genbacev et al., 1999; Smith et al., 1997). To clarify the mechanism of NRG1-dependent EVT formation, we assessed the effect of recombinant human NRG1 on cell survival. To this end, we treated placental floating explants with recombinant human NRG1 in the presence or absence of the topoisomerase I inhibitor camptothecin (CPT) and evaluated apoptosis rates by means of M30 CytoDEATH staining (Fig. 5A). In regard to the cell column, our quantifications showed that 5.4±1.4% of CCTs underwent apoptosis in the vehicle-treated control. Interestingly, treatment with recombinant human NRG1 alone was sufficient to profoundly decrease apoptosis rates below baseline levels. Upon addition of CPT, the rate of apoptosis was raised to 22.6±4.9% and significantly reduced in the presence of recombinant human NRG1. This NRG1-dependent protective effect was abolished upon antibody-mediated blockade of ErbB3. As expected, recombinant human NRG1 did not affect CPT-induced apoptosis of ErbB2^−^/ErbB3^−^ villous CTBs (Fig. 5B). In concert, we could show by western blotting that CPT-treated primary trophoblasts did respond to recombinant human NRG1 because they exhibited markedly diminished levels of cleaved caspase 3, its downstream target caspase-cleaved cytokeratin 18 (KRT18; recognized by the M30 antibody) and p53. This effect was again abrogated upon neutralization of ErbB3 (Fig. 5C). Taken together, these findings point to a pronounced anti-apoptotic effect of NRG1 on human trophoblasts.

### Suppression of apoptosis by NRG1 stabilizes the differentiated CCT population

**Fig. 6.**
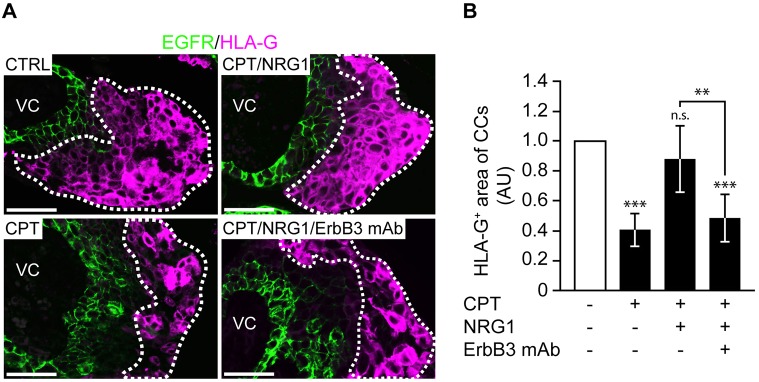
**NRG1-mediated suppression of apoptosis leads to the stabilization of trophoblast cell columns.** (A) First trimester placental floating explants were cultivated in the presence or absence of recombinant human NRG1 (20 ng/ml) and/or camptothecin (CPT, 1 µM) for 5 h. HLA-G^+^ areas of cell columns (CCs) are outlined in white. Where indicated, explants were pre-treated with a monoclonal ErbB3-blocking antibody (ErbB3 mAb, 10 µg/ml) for 30 min. Control (CTRL) treatment: 4 µM citrate solution, 10 µM DMSO and 10 µg/ml IgG mAb. Representative immunofluorescent co-staining for EGFR and HLA-G under each condition are shown. VC, villous core. Scale bars: 50 µm. (B) The HLA-G^+^ area of cell columns (CCs) was determined using Cell^P software. Data are expressed as mean±s.d. relative to vehicle control of four independent experiments. Statistical significance was determined with a one-way ANOVA test followed by Bonferroni's post-hoc correction. **P<0.01, ***P<0.001; n.s., not significant.

**Fig. 7.**
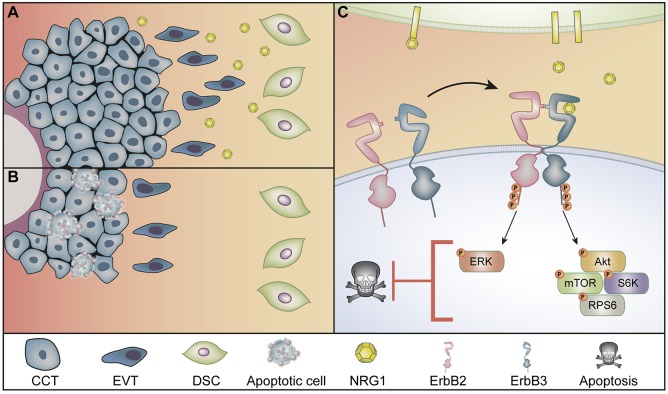
**Proposed model of NRG1 action at the foeto–maternal interface.** (A) Decidual stromal cells (DSCs) produce NRG1, which targets its bona fide receptor ErbB3 expressed by differentiated cell column trophoblasts (CCTs). The cellular response of NRG1 signalling involves the suppression of apoptotic cell death, which in turn leads to the stabilization of trophoblast cell column size. (B) When the NRG1–ErbB2–ErbB3 axis is disrupted or deregulated, trophoblasts are more susceptible to apoptotic stimuli and die from apoptosis. This leads to smaller cell columns, which in turn might result in insufficient extravillous trophoblast (EVT) invasion of the decidua. (C) Secreted NRG1 binds to the extracellular domain of ErbB3 and induces its heterodimerization with ErbB2. Following auto- and trans-phosphorylation of intracellular tyrosine residues, downstream effector kinases of the MAPK/ERK and PI3K–Akt pathways become activated.

To assess further the possibility that NRG1-dependent EVT formation results from protection against apoptotic cell death, we quantified the HLA-G^+^ cell column area of placental floating explants that had been treated with CPT and/or recombinant human NRG1 (Fig. 6A). In the presence of CPT, the HLA-G^+^ area of cell columns was decreased more than twofold when compared to that of the vehicle-treated control. Of note, recombinant human NRG1 inhibited CPT-induced apoptosis, and cell columns were comparable to healthy controls. Finally, when explant cultures were pre-incubated with the ErbB3-blocking antibody to counteract NRG1 signalling, cell columns were diminished in size, and values were similar to those of CPT treatment alone (Fig. 6B). Collectively, our data implicate NRG1-mediated ErbB2–ErbB3 signalling in the suppression of apoptosis, thereby protecting the pool of differentiated CCTs (Fig. 7).

## DISCUSSION

In this study, we show that decidua-derived NRG1 induces the formation of ErbB2–ErbB3 heterodimers in HLA-G^+^ trophoblast subtypes. Downstream signalling involves the activation of PI3K–Akt–S6K and MAPK/ERK pathways. Taken together, our functional data indicate that NRG1 promotes the survival of differentiated CCTs through suppression of apoptotic cell death. We further propose that the NRG1–ErbB2–ErbB3 axis is required for the maintenance of the distal cell column in order to ensure adequate decidual invasion by EVTs (Fig. 7).

The majority of data describing a functional role of ErbB2–ErbB3 heterodimers has been generated in mouse models of tumour progression. These efforts have led to the classification of this receptor complex as an oncogenic unit owing to the transforming potential of ErbB2 and ErbB3 when co-expressed (Holbro et al., 2003; Junttila et al., 2009; Zhang et al., 1996). In fact, the only data that are available to suggest a physiological function for this receptor combination demonstrate a pivotal role of NRG1-induced ErbB2–ErbB3 signalling during the development of the sympathetic nervous system in mice (Britsch et al., 1998). Interestingly, homozygous deletions of ErbB2, ErbB3 and NRG1 result in embryonic lethality, a phenotype often accompanied by placental defects (Meyer and Birchmeier, 1995; Natale et al., 2006; Riethmacher et al., 1997). Although the placenta has not been examined in ErbB2-, ErbB3- or NRG1-null mice, it is noteworthy that murine trophoblast giant cells, which are the equivalent to invasive EVTs in humans, have been found to express both ErbB2 and ErbB3 (Dackor et al., 2007; Lim et al., 1997). In addition, a recent study points to a role for ErbB2–ErbB3-mediated signalling at distant sites other than the nervous system, such as the upper spinous layers of the skin (Piepkorn et al., 2003). Well in line with our own published data (Fock et al., 2015), it seems obvious that during physiological processes, expression of ErbB2 and ErbB3 is restricted to differentiated non-dividing cell populations, most likely as a result of the transforming potential of ErbB2–ErbB3 heterodimers (Holbro et al., 2003). The results presented herein confirm that these two receptors colocalize in differentiated HLA-G^+^ trophoblasts and further demonstrate their ligand-induced dimerization. Interestingly, conditioned medium harvested from DSC cultures and recombinant human NRG1 induced identical phosphorylation patterns of ErbB2, ErbB3 and related downstream effectors – including ERK1/2, Akt1, mTOR, S6K and RPS6 – in primary trophoblasts. Moreover, receptor knockdown and neutralization experiments revealed that both ErbB2 and ErbB3 are involved in NRG1-mediated activation of Akt1 and ERK1/2. Taken together, these data pinpoint the formation of signalling-competent ErbB2–ErbB3 heterodimers in human trophoblasts.

Regarding the cellular source of NRG1, our results demonstrate that DSCs express and secrete the ligand *in situ* and *in vitro*, respectively. This finding is in accordance with a study reporting NRG1 expression in uterine glands and stromal cells of the non-pregnant endometrium (Srinivasan et al., 1999). In contrast, primary trophoblasts show no or very little expression of the ErbB3 ligand, contradicting autocrine effects. The *NRG1* gene encodes a huge variety of isoforms, which are produced through alternative splicing and promoter usage (Mei and Nave, 2014). Although these variants differ in their N-terminal sequence, they all share a conserved EGF-like domain that is responsible for receptor activation (Jones et al., 1998). Which particular NRG1 isoforms are expressed by DSCs awaits further investigation because the antibody used in this study was raised against the EGF-like domain. Owing to its expression as a transmembrane pro-form, activation of NRG1 requires proteolytic shedding by a disintegrin and metalloproteinase (ADAM), such as ADAM10, ADAM17 and ADAM19 or β-site amyloid precursor protein-cleaving enzyme (BACE)1 (Fleck et al., 2013; Luo et al., 2011; Yokozeki et al., 2007). Available data on NRG1-processing sheddases in the foeto–maternal interface indicate BACE1 expression in DSCs (Buhimschi et al., 2014) and associate EVTs with a strong upregulation of certain ADAM family members, including ADAM19 (Pollheimer et al., 2014). These data imply that NRG1 activation can potentially be triggered by DSCs in an intrinsic manner, or upon cell–cell contact with invasive EVTs through ADAM-mediated cleavage. Besides its expected localization at the cell membrane, NRG1 is also present within the nucleus of DSCs. Although not within the scope of this study, it seems likely that the NRG1 intracellular domain plays a role in DSC functions by acting as a transcriptional regulator, which has been reported previously in neurons (Bao et al., 2003).

To elucidate the biological effects of NRG1-induced ErbB2–ErbB3 signalling, we took advantage of various different human trophoblast model systems. Placental explants that had been maintained in serum-free medium or grown on collagen-I mimic EVT differentiation through the formation of proliferative cell columns, which give rise to invasive EVTs. These cultivation methods of placental explants allow the examination of trophoblast behaviour without the presence of multiple exogenous cytokines, growth factors and binding proteins that could potentially influence cell kinetics. Also, the spatial and ontological relationship between cells is maintained. In addition, primary trophoblast cultures comprising a pool of EGFR^+^ and HLA-G^+^ subtypes were used in this study. During the first 24 h of culture, primary trophoblasts cease proliferation and differentiate along the extravillous pathway, which is reflected by an increased expression of EVT marker genes (Biadasiewicz et al., 2014; Pollheimer et al., 2011). Our data indicate that NRG1 strongly promotes the formation of HLA-G^+^ cell columns in placental floating explants, as well as trophoblast outgrowth in explants grown on collagen-I. In addition, NRG1 enhances the expression of EVT-associated marker genes in outgrowing explants in an indirect manner, because gene transcription remained unaffected after short-term exposure to exogenous NRG1.

The vast majority of reports describe a pivotal function of ErbB2–ErbB3 heterodimers in cancer cell proliferation or motility (Holbro et al., 2003; Lyons et al., 2005). Unexpectedly, NRG1 did not influence these processes in any of the trophoblast model systems used. In this context, it is interesting to note that studies concentrating on ligand-independent ErbB2 or NRG1 signalling alone do suggest an additional role in cell survival (Andrechek et al., 2002; Zhao et al., 1998). Indeed, our present data indicate that NRG1 suppresses basal as well as CPT-induced apoptosis in CCTs. Consequently, we asked whether treatment with CPT negatively interferes with EVT formation and, if so, whether NRG1 inhibits this effect. Strikingly, we observed that NRG1 protects HLA-G^+^ trophoblasts from CPT-induced apoptosis, resulting in a significant stabilization of trophoblast cell columns. In agreement, placental explants treated with an ErbB3-blocking antibody underwent apoptosis owing to a lack of NRG1-mediated survival signals, which was also reflected by a remarkably decreased cell column area.

Development of the HLA-G^+^ EVT lineage involves a series of cellular events including mitogenesis, differentiation and the induction of an epithelial-to-mesenchymal transition, allowing for adequate trophoblast invasion into the decidua (Knofler and Pollheimer, 2013). In this regard, our findings support a novel concept bridging NRG1-dependent apoptotic resistance in ErbB2^+^/ErbB3^+^ CCTs with differentiation and EVT formation during early pregnancy. Downstream targets of the NRG1–ErbB2–ErbB3 axis include Akt1, mTOR, S6K and ERK1/2, all of which are related to survival in various cell types and model systems (Kennedy et al., 1997; Xia et al., 1995). Interestingly, a study using constitutively active viral (v)-ErbB demonstrates that the PI3K–Akt and MAPK/ERK pathways are equally important in ErbB-mediated survival (McCubrey et al., 2004). It has been further shown that only those cells, which rely on PI3K and MEK protein activity for proliferation, undergo apoptosis in the presence of PI3K and MEK inhibitors. Interestingly, our own recently published data suggest that activation of both Akt1 and ERK1/2 is instrumental for EGFR- and ErbB4-mediated proliferation in trophoblasts (Fock et al., 2015).

Collectively, our findings imply a vital role for tissue-resident DSCs in the paracrine control of trophoblast behaviour. Recent data substantiate this concept by demonstrating a supportive role of uterine natural killer (uNK) cells or uteroplacental macrophages in regulating trophoblast functions. uNK cells promote EVT invasion through the production of certain chemokines and growth factors (Hanna et al., 2006), whereas macrophages enhance placental growth through secretion of interleukin-33 (Fock et al., 2013). Although a well-balanced degree of apoptosis is likely to play a crucial role during trophoblast differentiation (Huppertz et al., 2006), there is evidence to suggest that enhanced rates of cell death in various trophoblast subtypes are indicative for pregnancy pathologies (Sharp et al., 2010). In this context, increased EVT-associated apoptosis has been noticed in sections of placental bed biopsies obtained from individuals with severe pre-eclampsia (DiFederico et al., 1999; Kadyrov et al., 2006). Another group postulates that outgrowing placental explants from high-resistance pregnancies are more susceptible to apoptotic stimuli than those from normal-resistance pregnancies *in vitro* (Whitley et al., 2007). In agreement, phenotypic alterations in different decidual cell types have been linked to placental pathologies. For example, certain combinations of EVT-specific HLA-C molecules and uNK cell-associated killer immunoglobulin-like receptor haplotypes greatly enhance the risk of developing pre-eclampsia (Hiby et al., 2004). Likewise, increased amounts of M1 macrophages seem to exert adverse effects such as the secretion of pro-inflammatory cytokines, a hallmark of pre-eclampsia (Schonkeren et al., 2011). Furthermore, a recent publication suggests that the impaired trophoblast phenotype noticed in pre-eclamptic individuals can, at least in part, be attributed to the uterine environment (Zhou et al., 2013). This conclusion was drawn upon the observation that pre-eclampsia-associated alterations in trophoblast gene expression return to control values after cultivating trophoblast cells for two days *in vitro*. In light of our data, it would be highly interesting to quantify apoptosis rates and to assess ErbB2 and ErbB3 expression in cell columns of pre-eclamptic placentas. Furthermore, it is tempting to speculate that a reduction of NRG1 activity might sensitize trophoblast cells to apoptosis. Hence, we propose that determination of NRG1 levels in DSCs from healthy and compromised pregnancies certainly deserves further attention in future research.

In conclusion, by showing that NRG1-dependent ErbB2–ErbB3 signalling is essential for the survival of differentiated trophoblast cell populations, we newly identify a physiological role for this cancer-associated receptor combination during human development.

## MATERIALS AND METHODS

### Tissue collection

First trimester placental (*n*=60) and decidual (*n*=9) specimens were obtained from elective terminations of viable pregnancies between the 6th and 12th gestational week. The gestational age was determined by ultrasound. Individuals were locally anaesthetized and treated with misoprostol before surgical intervention. Tissues were collected with written informed consent and utilization was approved by the Ethics Committee of the Medical University of Vienna. All investigations were conducted according to the principles expressed in the Declaration of Helsinki.

### Isolation of human primary trophoblasts

Primary trophoblasts were isolated from first trimester placental tissue as described previously (Fock et al., 2015). Briefly, placental villi were scraped and digested twice in 0.125% trypsin (Gibco^®^, Life Technologies) and 12.5 mg/ml DNase I (Sigma-Aldrich) for 30 min at 37°C. Pooled cell suspensions were filtered through a 100 μm cell strainer (Corning) and separated by using Percoll density gradient centrifugation (GE Healthcare Bio-Sciences, Uppsala, Sweden). Cells that had been collected from the 35–50% Percoll layer were incubated with red blood cell lysis buffer (155 mM NH_4_Cl, 10 mM KHCO_3_, 0.1 mM EDTA, pH 7.3) and plated on plastic dishes in DMEM/HAM's F12 medium (Gibco^®^) supplemented with 10% FCS (PAA Laboratories, 0.05 mg/ml gentamicin and 0.5 μg/ml fungizone (both from Gibco^®^) for 40 min to remove contaminating fibroblasts. Finally, trophoblasts were seeded onto fibronectin-coated (20 μg/ml; Millipore) dishes at a density of 5×10^5^ cells per well of a 24-well plate.

### Isolation of human primary DSCs

First trimester DSCs were isolated as described previously (Saleh et al., 2011). Briefly, decidual tissue was minced and digested three times in 2 mg/ml collagenase I (Gibco^®^) and 0.5 mg/ml DNase I for 20 min under agitation at 37°C. Dispersed cells were pooled and filtered through a 100 μm cell strainer. After red blood cell lysis, cells were plated onto plastic dishes and incubated for 24 h, followed by harvesting of serum-free conditioned medium and cell lysis. Samples were processed for RT-PCR and/or western blotting.

### Cell signalling

To investigate the activation of signalling mediators, overnight-cultivated primary trophoblasts were serum-starved for 4 h and stimulated with recombinant human NRG1 (20 ng/ml, Cell Signaling Technology) or DSC-conditioned medium for 5 and 20 min. When indicated, ErbB2 and ErbB3 single, or ErbB2–ErbB3 double knockdown was performed, or cells were pre-incubated with an anti-ErbB3 monoclonal antibody (10 µg/ml; R&D Systems) for 30 min. A citrate solution (4 µM) and an IgG mAb (10 µg/ml; Cell Signaling Technology) were used as vehicle control. Cells were lysed in Laemmli buffer and subjected to western blotting. Samples for the PathScan^®^ RTK Signaling Antibody Array Kit (Cell Signaling Technology) were processed according to the manufacturer's instructions. Signals were detected with chemiluminescence, and pixel intensities were determined by densitometry using Alpha View 3.1.1.0 software (Alpha Innotech, San Leandro, CA).

### Real-time PCR

Cells were lysed in peqGOLD TriFast™ (PEQLAB, Erlangen, Germany), and RNA was extracted according to the manufacturer's instructions. The RNA amount and purity were assessed with a ND-1000 NanoDrop spectrophotometer (PEQLAB). RNA was reverse transcribed with RevertAid H Minus Reverse Transcriptase (Thermo Scientific™, Life Technologies), and RT-PCR was performed using the 7500 Fast Real-Time PCR System (Applied Biosystems^®^, Life Technologies). The following FAM™-dye-labelled TaqMan^®^ MGB probes (Applied Biosystems^®^) were used: NRG1 (Hs00247620_m1), EGFR (Hs00193306_m1), HLA-G (Hs00365950_g1), TCF4 (Hs00181036_m1), ITGA1 (Hs00235006_m1), ITGA5 (Hs01547673_m1) and MMP2 (Hs00234422_m1). TATA-binding protein (4333769F) served as endogenous control. Relative mRNA expression was calculated with the comparative C_T_ method (Schmittgen and Livak, 2008).

### Co-immunoprecipitation and western blotting

Samples were lysed in Laemmli buffer and boiled for 5 min. Proteins were separated by using SDS-PAGE and blotted onto methanol-activated polyvinylidene difluoride membranes (GE Healthcare). After blocking in 5% non-fat dry milk in Tris-buffered saline containing 0.1% Tween-20, membranes were probed with the primary antibodies outlined in Table S1 overnight at 4°C, followed by incubation with horseradish-peroxidase-linked anti-mouse (1:25,000; GE Healthcare) or anti-rabbit (1:5000; Cell Signaling Technology) IgG secondary antibodies for 1 h at room temperature. The blots were developed with ECL Prime western blotting detection reagent (GE Healthcare), and proteins were visualized using MultiImage III FC Light Cabinet (Alpha Innotech) and Alpha View 3.1.1.0 software. For co-immunoprecipitation, approximately 5×10^6^ cells per condition were taken up in lysis buffer (catalogue number 9803, Cell Signaling Technology) supplemented with protease inhibitors (Sigma-Aldrich). The soluble fraction was incubated with a primary ErbB2 or ErbB3 antibody overnight at 4°C, and protein A agarose beads (catalogue number 9863, Cell Signaling Technology) were added for an additional 2 h. After five washing steps, samples were resuspended in Laemmli buffer and processed as described above.

### Immunofluorescent staining

First trimester placental and decidual tissues were fixed with 7.5% formaldehyde and embedded in paraffin (Merck). Serial sections were deparaffinised, and antigens were retrieved through boiling in PT Module Buffer 1 (pH 6; Thermo Scientific™) using a KOS MicrowaveStation (Milestone, Sorisole, Italy). After blocking in 0.05% fish skin gelatine (Sigma-Aldrich), sections were incubated with the primary antibodies outlined in Table S1 overnight at 4°C. Appropriate isotype-specific control antibodies were used accordingly. Subsequently, sections were incubated with goat anti-mouse or anti-rabbit IgG conjugated to Alexa-Fluor-488 or -546 (2 μg/ml; Molecular Probes^®^, Life Technologies) for 1 h at room temperature, counterstained with DAPI (1 μg/ml; Roche Diagnostics) and mounted in Fluoromount-G (SouthernBiotech). Images were acquired on a BX50 fluorescence microscope equipped with a CC12 digital camera and Cell^P software (Olympus, Hamburg, Germany).

### Flow cytometry

Single cell suspensions of primary overnight-cultivated trophoblasts were obtained by treating with Accutase (PAA Laboratories) for 5 min at 37°C. Cells were resuspended in ice-cold PBS containing 2% BSA (Sigma-Aldrich) and labelled with the FITC- and PE-conjugated antibodies outlined in Table S1 for 20 min at 4°C. Appropriate isotype-specific control antibodies were used accordingly. Data were acquired on a FACScan flow cytometer (BD Biosciences) and analyzed with FlowJo 7.6.4 software (Tree Star, Ashland, OR).

### siRNA transfection

Freshly isolated primary trophoblasts were transfected with SMARTpool^®^ ON-TARGETplus siRNAs (GE Dharmacon) using Lipofectamine^®^ RNAiMAX reagent (Invitrogen™, Life Technologies) according to the manufacturer's instructions. The following siRNAs against the indicated proteins were used at a concentration of 40 nM: ErbB2 (L-003126-00-0005), ErbB3 (L-003127-00-0005) and non-targeting control (D-001810-10-05). After 48 h, transfected cells were subjected to western blotting.

### EVT formation assay

Placental villi (7–9th gestational week) were dissected and placed on collagen-I drops (Corning), as described previously (Biadasiewicz et al., 2014). After 5 h, placental explants were covered with DMEM/Ham's F-12 medium supplemented with 0.05 mg/ml gentamicin and further incubated for 24 h in the presence or absence of recombinant human NRG1 (2, 20 or 200 ng/ml). A citrate solution (4 µM) was used as vehicle control. The area of outgrowth was digitally photographed on a BX50 fluorescence microscope and quantified using Cell^P software. RNA was extracted by lysing explants in peqGOLD TriFast™ subsequent to the removal of anchoring villi. Pooled RNA of control- or recombinant-human-NRG1-treated trophoblast cell columns was subjected to RT-PCR for monitoring the influence of recombinant human NRG1 on EVT marker expression. Because EGFR^+^ CCTs are non-responsive to NRG1 owing to a lack of ErbB3 expression, gene expression levels were normalized to EGFR mRNA values (set to 1).

### CPT-induced apoptosis

Primary trophoblasts and dissected placental villi (7–9th gestational week, *n*=15 per condition) were cultivated in DMEM/Ham's F-12 medium supplemented with 0.05 mg/ml gentamicin for 24 h. Placental villi were selected for the presence of attached cell columns under a light microscope. Subsequently, trophoblast cells or floating explants were pre-incubated with an ErbB3 mAb (10 µg/ml) for 30 min, followed by the addition of recombinant human NRG1 (20 ng/ml) and treatment with CPT (1 µM; MP Biomedicals, Santa Ana, CA) for 5 h, before being processed for western blotting or immunofluorescent staining, respectively. A citrate solution (4 µM), DMSO (10 µM) and an IgG mAb (10 µg/ml) were used as vehicle control.

### Statistical analyses

All data are expressed as mean±s.d. Statistical analyses were conducted using GraphPad Prism 6 (GraphPad Software). For direct comparisons, a two-tailed unpaired Student's *t*-test was used. Analysis of more than two groups was performed using a one-way ANOVA followed by Bonferroni's post-hoc correction. The influence of recombinant human NRG1 on the expression of EVT marker genes was determined by using a two-way ANOVA test followed by Šidák's post-hoc correction. A *P*-value lower than 0.05 was considered statistically significant.

## References

[JCS176933C1] Andrechek E. R., Hardy W. R., Girgis-Gabardo A. A., Perry R. L. S., Butler R., Graham F. L., Kahn R. C., Rudnicki M. A. and Muller W. J. (2002). ErbB2 is required for muscle spindle and myoblast cell survival. *Mol. Cell. Biol.* 22, 4714-4722. 10.1128/MCB.22.13.4714-4722.2002 12052879 PMC133917

[JCS176933C2] Bao J., Wolpowitz D., Role L. W. and Talmage D. A. (2003). Back signaling by the Nrg-1 intracellular domain. *J. Cell Biol.* 161, 1133-1141. 10.1083/jcb.200212085 12821646 PMC2172983

[JCS176933C3] Biadasiewicz K., Fock V., Dekan S., Proestling K., Velicky P., Haider S., Knofler M., Frohlich C. and Pollheimer J. (2014). Extravillous trophoblast-associated ADAM12 exerts pro-invasive properties, including induction of integrin beta 1-mediated cellular spreading. *Biol. Reprod.* 90, 101 10.1095/biolreprod.113.115279 24695627

[JCS176933C4] Britsch S., Li L., Kirchhoff S., Theuring F., Brinkmann V., Birchmeier C. and Riethmacher D. (1998). The ErbB2 and ErbB3 receptors and their ligand, neuregulin-1, are essential for development of the sympathetic nervous system. *Genes Dev.* 12, 1825-1836. 10.1101/gad.12.12.1825 9637684 PMC316903

[JCS176933C5] Brosens I. A., Robertson W. B. and Dixon H. G. (1970). The role of the spiral arteries in the pathogenesis of pre-eclampsia. *J. Pathol.* 101, Pvi. 5504740

[JCS176933C6] Brosens I., Dixon H. G. and Robertson W. B. (1977). Fetal growth retardation and the arteries of the placental bed. *Br. J. Obstet. Gynaecol.* 84, 656-663. 10.1111/j.1471-0528.1977.tb12676.x 911717

[JCS176933C7] Buhimschi I. A., Nayeri U. A., Zhao G., Shook L. L., Pensalfini A., Funai E. F., Bernstein I. M., Glabe C. G. and Buhimschi C. S. (2014). Protein misfolding, congophilia, oligomerization, and defective amyloid processing in preeclampsia. *Sci. Transl. Med.* 6, 245ra92 10.1126/scitranslmed.3008808 25031267

[JCS176933C8] Citri A. and Yarden Y. (2006). EGF-ERBB signalling: towards the systems level. *Nat. Rev. Mol. Cell Biol.* 7, 505-516. 10.1038/nrm1962 16829981

[JCS176933C9] Dackor J., Strunk K. E., Wehmeyer M. M. and Threadgill D. W. (2007). Altered trophoblast proliferation is insufficient to account for placental dysfunction in Egfr null embryos. *Placenta* 28, 1211-1218. 10.1016/j.placenta.2007.07.005 17822758 PMC2121666

[JCS176933C10] Damsky C. H., Fitzgerald M. L. and Fisher S. J. (1992). Distribution patterns of extracellular matrix components and adhesion receptors are intricately modulated during first trimester cytotrophoblast differentiation along the invasive pathway, in vivo. *J. Clin. Invest.* 89, 210-222. 10.1172/JCI115565 1370295 PMC442839

[JCS176933C11] DiFederico E., Genbacev O. and Fisher S. J. (1999). Preeclampsia is associated with widespread apoptosis of placental cytotrophoblasts within the uterine wall. *Am. J. Pathol.* 155, 293-301. 10.1016/S0002-9440(10)65123-1 10393861 PMC1866652

[JCS176933C12] Ellis S. A., Palmer M. S. and McMichael A. J. (1990). Human trophoblast and the choriocarcinoma cell line BeWo express a truncated HLA Class I molecule. *J. Immunol.* 144, 731-735. 2295808

[JCS176933C13] Falls D. L. (2003). Neuregulins: functions, forms, and signaling strategies. *Exp. Cell Res.* 284, 14-30. 10.1016/S0014-4827(02)00102-7 12648463

[JCS176933C14] Fisher S. J., Cui T. Y., Zhang L., Hartman L., Grahl K., Zhang G. Y., Tarpey J. and Damsky C. H. (1989). Adhesive and degradative properties of human placental cytotrophoblast cells in vitro. *J. Cell Biol.* 109, 891-902. 10.1083/jcb.109.2.891 2474556 PMC2115717

[JCS176933C15] Fleck D., van Bebber F., Colombo A., Galante C., Schwenk B. M., Rabe L., Hampel H., Novak B., Kremmer E., Tahirovic S. et al. (2013). Dual cleavage of neuregulin 1 type III by BACE1 and ADAM17 liberates its EGF-like domain and allows paracrine signaling. *J. Neurosci.* 33, 7856-7869. 10.1523/JNEUROSCI.3372-12.2013 23637177 PMC6618983

[JCS176933C16] Fock V., Mairhofer M., Otti G. R., Hiden U., Spittler A., Zeisler H., Fiala C., Knofler M. and Pollheimer J. (2013). Macrophage-derived IL-33 is a critical factor for placental growth. *J. Immunol.* 191, 3734-3743. 10.4049/jimmunol.1300490 23997215

[JCS176933C17] Fock V., Plessl K., Fuchs R., Dekan S., Milla S. K., Haider S., Fiala C., Knofler M. and Pollheimer J. (2015). Trophoblast subtype-specific EGFR/ERBB4 expression correlates with cell cycle progression and hyperplasia in complete hydatidiform moles. *Hum. Reprod.* 30, 789-799. 10.1093/humrep/dev027 25740878

[JCS176933C18] Garrett T. P. J., McKern N. M., Lou M., Elleman T. C., Adams T. E., Lovrecz G. O., Kofler M., Jorissen R. N., Nice E. C., Burgess A. W. et al. (2003). The crystal structure of a truncated ErbB2 ectodomain reveals an active conformation, poised to interact with other ErbB receptors. *Mol. Cell* 11, 495-505. 10.1016/S1097-2765(03)00048-0 12620236

[JCS176933C19] Genbacev O., Schubach S. A. and Miller R. K. (1992). Villous culture of first trimester human placenta--model to study extravillous trophoblast (EVT) differentiation. *Placenta* 13, 439-461. 10.1016/0143-4004(92)90051-T 1470605

[JCS176933C20] Genbacev O., DiFederico E., McMaster M. and Fisher S. J. (1999). Invasive cytotrophoblast apoptosis in pre-eclampsia. *Hum. Reprod.* 14 Suppl. 2, 59-66. 10.1093/humrep/14.suppl_2.59 10690801

[JCS176933C21] Guy P. M., Platko J. V., Cantley L. C., Cerione R. A. and Carraway K. L. III (1994). Insect cell-expressed p180erbB3 possesses an impaired tyrosine kinase activity. *Proc. Natl. Acad. Sci. USA* 91, 8132-8136. 10.1073/pnas.91.17.8132 8058768 PMC44559

[JCS176933C22] Hanna J., Goldman-Wohl D., Hamani Y., Avraham I., Greenfield C., Natanson-Yaron S., Prus D., Cohen-Daniel L., Arnon T. I., Manaster I. et al. (2006). Decidual NK cells regulate key developmental processes at the human fetal-maternal interface. *Nat. Med.* 12, 1065-1074. 10.1038/nm1452 16892062

[JCS176933C23] Hiby S. E., Walker J. J., O'Shaughnessy K. M., Redman C. W. G., Carrington M., Trowsdale J. and Moffett A. (2004). Combinations of maternal KIR and fetal HLA-C genes influence the risk of preeclampsia and reproductive success. *J. Exp. Med.* 200, 957-965. 10.1084/jem.20041214 15477349 PMC2211839

[JCS176933C24] Holbro T., Beerli R. R., Maurer F., Koziczak M., Barbas C. F. III and Hynes N. E. (2003). The ErbB2/ErbB3 heterodimer functions as an oncogenic unit: ErbB2 requires ErbB3 to drive breast tumor cell proliferation. *Proc. Natl. Acad. Sci. USA* 100, 8933-8938. 10.1073/pnas.1537685100 12853564 PMC166416

[JCS176933C25] Huppertz B., Kadyrov M. and Kingdom J. C. P. (2006). Apoptosis and its role in the trophoblast. *Am. J. Obstet. Gynecol.* 195, 29-39. 10.1016/j.ajog.2005.07.039 16579915

[JCS176933C26] Jones J. T., Ballinger M. D., Pisacane P. I., Lofgren J. A., Fitzpatrick V. D., Fairbrother W. J., Wells J. A. and Sliwkowski M. X. (1998). Binding interaction of the heregulinbeta egf domain with ErbB3 and ErbB4 receptors assessed by alanine scanning mutagenesis. *J. Biol. Chem.* 273, 11667-11674. 10.1074/jbc.273.19.11667 9565587

[JCS176933C27] Junttila T. T., Akita R. W., Parsons K., Fields C., Lewis Phillips G. D., Friedman L. S., Sampath D. and Sliwkowski M. X. (2009). Ligand-independent HER2/HER3/PI3K complex is disrupted by trastuzumab and is effectively inhibited by the PI3K inhibitor GDC-0941. *Cancer Cell* 15, 429-440. 10.1016/j.ccr.2009.03.020 19411071

[JCS176933C28] Kadyrov M., Kingdom J. C. P. and Huppertz B. (2006). Divergent trophoblast invasion and apoptosis in placental bed spiral arteries from pregnancies complicated by maternal anemia and early-onset preeclampsia/intrauterine growth restriction. *Am. J. Obstet. Gynecol.* 194, 557-563. 10.1016/j.ajog.2005.07.035 16458661

[JCS176933C29] Kennedy S. G., Wagner A. J., Conzen S. D., Jordan J., Bellacosa A., Tsichlis P. N. and Hay N. (1997). The PI 3-kinase/Akt signaling pathway delivers an anti-apoptotic signal. *Genes Dev.* 11, 701-713. 10.1101/gad.11.6.701 9087425

[JCS176933C30] Knofler M. and Pollheimer J. (2013). Human placental trophoblast invasion and differentiation: a particular focus on Wnt signaling. *Front. Genet.* 4, 190 10.3389/fgene.2013.00190 24133501 PMC3783976

[JCS176933C31] Lim H., Dey S. K. and Das S. K. (1997). Differential expression of the erbB2 gene in the periimplantation mouse uterus: potential mediator of signaling by epidermal growth factor-like growth factors. *Endocrinology* 138, 1328-1337. 9048643 10.1210/endo.138.3.4991

[JCS176933C32] Luo X., Prior M., He W., Hu X., Tang X., Shen W., Yadav S., Kiryu-Seo S., Miller R., Trapp B. D. et al. (2011). Cleavage of neuregulin-1 by BACE1 or ADAM10 protein produces differential effects on myelination. *J. Biol. Chem.* 286, 23967-23974. 10.1074/jbc.M111.251538 21576249 PMC3129178

[JCS176933C33] Lyons D. A., Pogoda H.-M., Voas M. G., Woods I. G., Diamond B., Nix R., Arana N., Jacobs J. and Talbot W. S. (2005). erbb3 and erbb2 are essential for schwann cell migration and myelination in zebrafish. *Curr. Biol.* 15, 513-524. 10.1016/j.cub.2005.02.030 15797019

[JCS176933C34] McCubrey J. A., Shelton J. G., Steelman L. S., Franklin R. A., Sreevalsan T. and McMahon M. (2004). Effects of a conditionally active v-ErbB and an EGF-R inhibitor on transformation of NIH-3T3 cells and abrogation of cytokine dependency of hematopoietic cells. *Oncogene* 23, 7810-7820. 10.1038/sj.onc.1208055 15361836

[JCS176933C35] Mei L. and Nave K.-A. (2014). Neuregulin-ERBB signaling in the nervous system and neuropsychiatric diseases. *Neuron* 83, 27-49. 10.1016/j.neuron.2014.06.007 24991953 PMC4189115

[JCS176933C36] Mei L. and Xiong W.-C. (2008). Neuregulin 1 in neural development, synaptic plasticity and schizophrenia. *Nat. Rev. Neurosci.* 9, 437-452. 10.1038/nrn2392 18478032 PMC2682371

[JCS176933C37] Meyer D. and Birchmeier C. (1995). Multiple essential functions of neuregulin in development. *Nature* 378, 386-390. 10.1038/378386a0 7477375

[JCS176933C38] Moffett A. and Loke C. (2006). Immunology of placentation in eutherian mammals. *Nat. Rev. Immunol.* 6, 584-594. 10.1038/nri1897 16868549

[JCS176933C39] Natale D. R., Starovic M. and Cross J. C. (2006). Phenotypic analysis of the mouse placenta. *Methods Mol. Med.* 121, 275-293. 16251749 10.1385/1-59259-983-4:273

[JCS176933C40] Piepkorn M., Predd H., Underwood R. and Cook P. (2003). Proliferation-differentiation relationships in the expression of heparin-binding epidermal growth factor-related factors and erbB receptors by normal and psoriatic human keratinocytes. *Arch. Dermatol. Res.* 295, 93-101. 10.1007/s00403-003-0391-x 12768307

[JCS176933C41] Pijnenborg R., Dixon G., Robertson W. B. and Brosens I. (1980). Trophoblastic invasion of human decidua from 8 to 18 weeks of pregnancy. *Placenta* 1, 3-19. 10.1016/S0143-4004(80)80012-9 7443635

[JCS176933C42] Pollheimer J., Loregger T., Sonderegger S., Saleh L., Bauer S., Bilban M., Czerwenka K., Husslein P. and Knofler M. (2006). Activation of the canonical wingless/T-cell factor signaling pathway promotes invasive differentiation of human trophoblast. *Am. J. Pathol.* 168, 1134-1147. 10.2353/ajpath.2006.050686 16565489 PMC1606554

[JCS176933C43] Pollheimer J., Haslinger P., Fock V., Prast J., Saleh L., Biadasiewicz K., Jetne-Edelmann R., Haraldsen G., Haider S., Hirtenlehner-Ferber K. et al. (2011). Endostatin suppresses IGF-II-mediated signaling and invasion of human extravillous trophoblasts. *Endocrinology* 152, 4431-4442. 10.1210/en.2011-1196 21933871

[JCS176933C44] Pollheimer J., Fock V. and Knofler M. (2014). Review: the ADAM metalloproteinases - novel regulators of trophoblast invasion? *Placenta* 35 Suppl., S57-S63. 10.1016/j.placenta.2013.10.012 24231445

[JCS176933C45] Riethmacher D., Sonnenberg-Riethmacher E., Brinkmann V., Yamaai T., Lewin G. R. and Birchmeier C. (1997). Severe neuropathies in mice with targeted mutations in the ErbB3 receptor. *Nature* 389, 725-730. 10.1038/39593 9338783

[JCS176933C46] Saleh L., Otti G. R., Fiala C., Pollheimer J. and Knofler M. (2011). Evaluation of human first trimester decidual and telomerase-transformed endometrial stromal cells as model systems of in vitro decidualization. *Reprod. Biol. Endocrinol.* 9, 155 10.1186/1477-7827-9-155 22151839 PMC3267678

[JCS176933C47] Schmittgen T. D. and Livak K. J. (2008). Analyzing real-time PCR data by the comparative C(T) method. *Nat. Protoc.* 3, 1101-1108. 10.1038/nprot.2008.73 18546601

[JCS176933C48] Schonkeren D., van der Hoorn M.-L., Khedoe P., Swings G., van Beelen E., Claas F., van Kooten C., de Heer E. and Scherjon S. (2011). Differential distribution and phenotype of decidual macrophages in preeclamptic versus control pregnancies. *Am. J. Pathol.* 178, 709-717. 10.1016/j.ajpath.2010.10.011 21281803 PMC3069820

[JCS176933C49] Sharp A. N., Heazell A. E. P., Crocker I. P. and Mor G. (2010). Placental apoptosis in health and disease. *Am. J. Reprod. Immunol.* 64, 159-169. 10.1111/j.1600-0897.2010.00837.x 20367628 PMC3025811

[JCS176933C50] Smith S. C., Baker P. N. and Symonds E. M. (1997). Increased placental apoptosis in intrauterine growth restriction. *Am. J. Obstet. Gynecol.* 177, 1395-1401. 10.1016/S0002-9378(97)70081-4 9423741

[JCS176933C51] Srinivasan R., Benton E., McCormick F., Thomas H. and Gullick W. J. (1999). Expression of the c-erbB-3/HER-3 and c-erbB-4/HER-4 growth factor receptors and their ligands, neuregulin-1 alpha, neuregulin-1 beta, and betacellulin, in normal endometrium and endometrial cancer. *Clin. Cancer Res.* 5, 2877-2883. 10537356

[JCS176933C52] Ursini-Siegel J., Schade B., Cardiff R. D. and Muller W. J. (2007). Insights from transgenic mouse models of ERBB2-induced breast cancer. *Nat. Rev. Cancer* 7, 389-397. 10.1038/nrc2127 17446858

[JCS176933C53] Whitley G. S. J., Dash P. R., Ayling L.-J., Prefumo F., Thilaganathan B. and Cartwright J. E. (2007). Increased apoptosis in first trimester extravillous trophoblasts from pregnancies at higher risk of developing preeclampsia. *Am. J. Pathol.* 170, 1903-1909. 10.2353/ajpath.2007.070006 17525258 PMC1899436

[JCS176933C54] Xia Z., Dickens M., Raingeaud J., Davis R. J. and Greenberg M. E. (1995). Opposing effects of ERK and JNK-p38 MAP kinases on apoptosis. *Science* 270, 1326-1331. 10.1126/science.270.5240.1326 7481820

[JCS176933C55] Yokozeki T., Wakatsuki S., Hatsuzawa K., Black R. A., Wada I. and Sehara-Fujisawa A. (2007). Meltrin beta (ADAM19) mediates ectodomain shedding of Neuregulin beta1 in the Golgi apparatus: fluorescence correlation spectroscopic observation of the dynamics of ectodomain shedding in living cells. *Genes Cells* 12, 329-343. 10.1111/j.1365-2443.2007.01060.x 17352738

[JCS176933C56] Zhang K., Sun J., Liu N., Wen D., Chang D., Thomason A. and Yoshinaga S. K. (1996). Transformation of NIH 3T3 cells by HER3 or HER4 receptors requires the presence of HER1 or HER2. *J. Biol. Chem.* 271, 3884-3890. 10.1074/jbc.271.7.3884 8632008

[JCS176933C57] Zhao Y.-Y., Sawyer D. R., Baliga R. R., Opel D. J., Han X., Marchionni M. A. and Kelly R. A. (1998). Neuregulins promote survival and growth of cardiac myocytes: persistence of ErbB2 and ErbB4 expression in neonatal and adult ventricular myocytes. *J. Biol. Chem.* 273, 10261-10269. 10.1074/jbc.273.17.10261 9553078

[JCS176933C58] Zhou Y., Gormley M. J., Hunkapiller N. M., Kapidzic M., Stolyarov Y., Feng V., Nishida M., Drake P. M., Bianco K., Wang F. et al. (2013). Reversal of gene dysregulation in cultured cytotrophoblasts reveals possible causes of preeclampsia. *J. Clin. Invest.* 123, 2862-2872. 10.1172/JCI66966 23934129 PMC3999620

